# Prevalence of Oral Alterations and Correlation Between Oral and Cutaneous Neurofibromas in Neurofibromatosis Type 1: A Retrospective Case–Control Study

**DOI:** 10.1111/jop.70056

**Published:** 2025-09-17

**Authors:** Pâmella de Pinho Montovani, Gabriela Pizão Werneck Moreira da Costa, Rafaela Elvira Rozza‐de‐Menezes, Karin Soares Cunha

**Affiliations:** ^1^ Graduate Program in Pathology School of Medicine, Universidade Federal Fluminense Niterói Brazil; ^2^ Department of Pathology, School of Medicine Universidade Federal Fluminense Niterói Brazil; ^3^ Neurofibromatosis National Center (Centro Nacional de Neurofibromatose, CNNF) Rio de Janeiro Brazil

**Keywords:** cutaneous neurofibroma, neurofibromatosis 1, oral alterations, oral neurofibroma

## Abstract

**Objective:**

The aim of this study was to determine the prevalence of oral alterations detectable through physical examination in NF1 individuals. Additionally, we assessed the correlation between the number of oral and cutaneous neurofibromas.

**Design:**

This retrospective study evaluated oral alterations in individuals with and without NF1. In the NF1 group, associations between oral and cutaneous neurofibromas, age, sex, pregnancy, and family history of NF1 were assessed.

**Results:**

A total of 327 participants were evaluated (81 with NF1 and 246 controls). Oral mucosal alterations (92.6% vs. 79.3%) and exostoses (12.3% vs. 4.5%) were significantly more prevalent in the NF1 group. The most frequent oral alterations were enlarged fungiform papillae (46.9% vs. 8.1%), coated tongue (45.7% vs. 29.3%), neurofibromas (38.3% versus none), physiological melanin pigmentation (30.9% vs. 10.6%), and exostoses (12.3% vs. 4.5%). Oral neurofibromas were more prevalent in older individuals, without a family history of NF1, and those with multiple cutaneous neurofibromas.

**Conclusions:**

Enlarged fungiform papillae and oral neurofibromas are the most common oral alterations detectable through physical examination in NF1. Coated tongue, physiological melanin pigmentation, and exostoses were also more frequent in NF1. The number of oral and cutaneous neurofibromas is correlated. These findings underscore the necessity of regular oral assessments in NF1 individuals.

## 1 Introduction

Neurofibromatosis type 1 (NF1) is a common genetic disorder with frequent oral involvement [1]. Enlarged fungiform papillae and oral neurofibromas are the most common findings reported in the literature [2], but large‐scale evaluations remain scarce. Cutaneous neurofibromas affect ~99% of patients, with some developing hundreds or thousands [3], whereas multiple oral neurofibromas are uncommon. We hypothesized that NF1 individuals have additional underrecognized oral alterations and that oral neurofibroma burden correlates with cutaneous tumor counts. We evaluated the prevalence and pattern of oral alterations in NF1 and their relationship with cutaneous neurofibromas.

## 2 Material and Methods

This retrospective case–control study was conducted at the Oral Diagnosis Outpatient Clinic, Antônio Pedro University Hospital, Brazil (Ethics approval #4.780.584). The NF1 group comprised 81 participants diagnosed according to the revised criteria [1]. Controls (*n* = 246) were randomly selected from the same database, excluding NF1 or other genetic/systemic conditions affecting oral health.

Photographic documentation and cytopathological examination of the entire oral mucosa, regardless of visible lesions, was recorded in medical files. As NF1 patients had a mean of 2.9 clinical appointments, data from the first three control appointments were analyzed.

Variables for both groups included age, sex, self‐reported skin color, enlarged fungiform papillae (from tongue photographs), other oral alterations, cytopathology results, and denture use. For NF1, additional variables included family history, pregnancies (females), and cutaneous neurofibroma counts using paper frames [4].

Oral candidiasis was diagnosed clinically and cytopathologically; subclinical cases were defined by positive cytology without clinical signs [5]. Suspected oral neurofibromas were biopsied when possible; histopathology and S100 immunohistochemistry confirmed diagnosis. Imaging studies were not included.

Statistical analysis used the Shapiro–Wilk test, chi‐square or Fisher's exact tests, *t*‐test with Cohen's *d* or Mann–Whitney *U*, and Spearman's correlation. Chi‐square was applied only when ≥ 10% of NF1 participants were affected. LOESS modeled the relationship between oral and cutaneous neurofibromas.

## 3 Results

Table 1 presents demographic data, and Table 2 compares oral lesion prevalence in NF1 and controls. Oral mucosal alterations (92.6% vs. 79.3%; *p* = 0.006) and exostoses (12.3% vs. 4.5%; *p* = 0.012) were more prevalent in NF1.

**TABLE 1:** Demographic data of neurofibromatosis 1 and control groups.

Demographic data	NF1 (*n* = 81)	Controls (*n* = 246)	*p*
Sex			
Male	24 (29.6%)	96 (39%)	0.128^a^
Female	57 (70.4%)	150 (61%)	
Age (years)			
Mean (±SD)	39.7 (±16.5)	53.5 (±17.7)	< **0.0001** ^b^
Minimum	3	0.7	
Maximum	75	85	

Age groups (years)	*n* (%)	*n* (%)	
0–10	3 (3.7%)	10 (4.1%)	—
11–20	9 (11.1%)	8 (3.3%)	
21–30	10 (12.2%)	13 (5.3%)	
31–40	13 (16%)	13 (5.3%)	
41–50	24 (29.6%)	38 (15.4%)	
51–60	11 (13.6%)	63 (25.6%)	
61–70	6 (7.4%)	69 (28%)	
71–80	2 (2.5%)	28 (11.4%)	
81–90	0 (0%)	2 (0.8%)	
No information	3 (3.7%)	2 (0.8%)	

Skin color	*n* (%)	*n* (%)	
Black	51 (62.9%)	138 (56%)	0.892^a^
White	22 (27.2%)	91 (37%)	
No information	8 (9.9%)	17 (6.9%)	

*Note*: Bold results indicate statistically significant.

Abbreviations: NF1, neurofibromatosis type 1; SD, standard deviation.

^a^ Pearson's chi‐square.

^b^ Mann–Whitney test.

**TABLE 2:** Prevalence and statistical analysis of oral alterations in neurofibromatosis type 1 and controls.

Oral alterations	NF1 (*n* = 81)	Controls (*n* = 246)	*p* ^a^	Odds ratio (IC 95%)
Soft tissue (total)	75 (92.6%)	195 (79.3%)	**0.006**	0.306 (0.126–0.743)
Exostoses (total)	10 (12.3%)	11 (4.5%)	**0.012**	0.332 (0.136–0.815)
Enlarged fungiform papillae	38 (46.9%)	20 (8.1%)	**< 0.0001**	0.100 (0.053–0.188)
Coated tongue	37 (45.7%)	72 (29.3%)	**0.007**	0.492 (0.294–0.825)
Neurofibromas	31 (38.3%)	None (—)	**< 0.0001**	—
Physiological melanin pigmentation	25 (30.9%)	26 (10.6%)	**< 0.0001**	0.265 (0.142–0.493)
Fissured tongue	20 (24.7%)	52 (21.1%)	0.503	0.818 (0.453–1.476)
Candidiasis	17/57 (29.8%)	80 (32.5%)	0.694	1.34 (0.606–2.123)
Geographic tongue	7 (8.6%)	22 (8.9%)	—^b^	—^b^
Crenated tongue	5 (6.2%)	14 (5.7%)	—^b^	—^b^
Fordyce granules	4 (4.9%)	16 (6.5%)	—^b^	—^b^
Unspecific ulcer^c^	4 (4.9%)	17 (6.9%)	—^b^	—^b^
Ankyloglossia	3 (3.7%)	None (—)	—^b^	—^b^
Recurrent herpes simplex lesion	2 (2.5%)	6 (2.4%)	—^b^	—^b^
Leukoedema	2 (2.5%)	5 (2%)	—^b^	—^b^
Lichenoid reaction	2 (2.5%)	2 (0.8%)	—^b^	—^b^
Enlarged fimbriated fold of tongue	2 (2.5%)	None (—)	—^b^	—^b^
Inflammatory fibrous hyperplasia	1 (1.2%)	8 (3.3%)	—^b^	—^b^
Unspecific petechiae^c^	1 (1.2%)	None (—)	—^b^	—^b^
Oral condyloma acuminatum	1 (1.2%)	3 (1.2%)	—^b^	—^b^
Morsicatio buccarum	1 (1.2%)	5 (2%)	—^b^	—^b^
Focal fibrous hyperplasia	1 (1.2%)	9 (3.7%)	—^b^	—^b^
Actinic cheilitis	1 (1.2%)	26 (10.6%)	—^b^	—^b^
Fibrolipoma	1 (1.2%)	3 (1.2%)	—^b^	—^b^

Abbreviations: IC, interval of confidence; NF1, neurofibromatosis type 1.

^a^ Pearson's chi‐square test.

^b^ Inferential statistics were not applied in oral alterations prevalence of less than 10% of the participants with neurofibromatosis type 1.

^c^ Alterations without description of the etiological factor.

Enlarged fungiform papillae was the most frequent alteration, affecting 46.9% of NF1 vs. 8.1% of controls (*p* < 0.0001), and was more common in younger participants (mean = 33.7 ± 17.4 years; *p* = 0.002). Coated tongue, the second most common alteration, affected 45.7% of NF1 vs. 29.3% of controls (*p* = 0.007).

**FIGURE 1:**
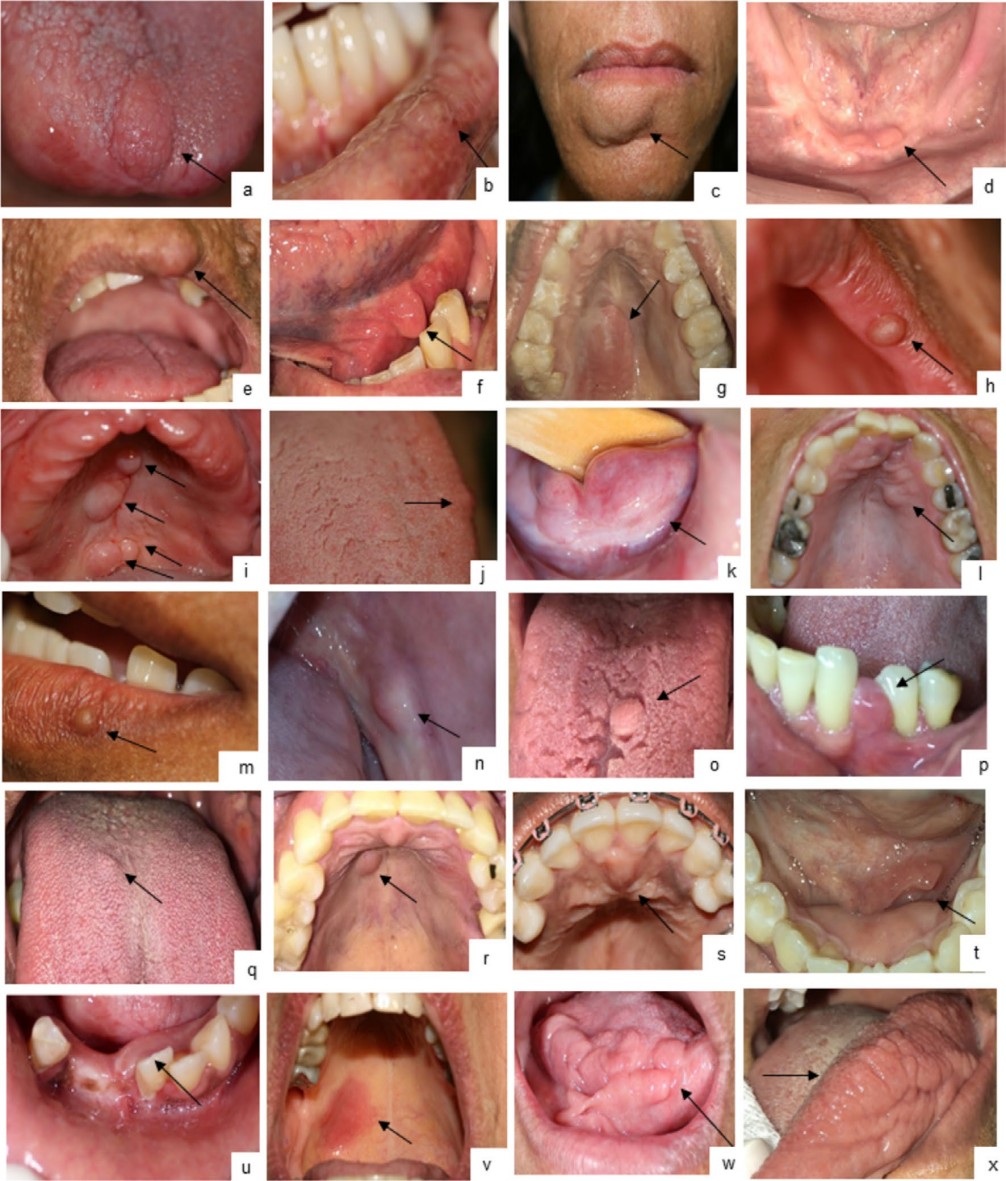
Clinical presentation of suspected and histopathologically confirmed oral neurofibromas in NF1 participants. (a) Nodule on apex of tongue; (b) Papule on inferior labial mucosa; (c) Nodule on lower lip, right side; (d) Papule on anterior inferior alveolar ridge; (e) Nodule on upper lip, left side; (f) Nodule on floor of mouth, left side; (g) Nodule on hard palate mucosa; (h) Papule on upper lip semimucosa, left side; (i) Four nodules on hard and soft palate mucosa; (j) Papule on tongue dorsum; (k) Diffuse nodule on floor of mouth; (l) Nodule on hard palate mucosa, left side; (m)* Papule on lower lip semimucosa, left side; (n)* Nodule on buccal mucosa, left side; (o)* Nodule on tongue dorsum; (p)* Nodule on interdental gingiva, left side; (q) Papule on tongue dorsum; (r) Nodule on hard palate mucosa, right side; (s) Nodule on incisive papilla; (t) Diffuse swelling on floor of mouth, left side; (u)* Diffuse swelling on free and attached gingiva; (v)* Erythematous macule on hard and soft palate mucosa, right side; (w) Hemimacroglossia, right side; (x) Hemimacroglossia, left side. *Indicates that the lesions were confirmed through histopathological examination.

Oral neurofibromas occurred exclusively in NF1 and ranked third in frequency (38.3%). Most were localized (97.2%; mean = 2.48 ± 2.5; range 1–13); 17.3% had multiple lesions, 20.9% single tumors. Lesions were usually asymptomatic, normochromic papules/nodules (90%), with occasional diffuse swellings (5.7%) and one pruritic erythematous macule (Figure 1). The palate was the most frequent site (35.7%), followed by tongue (24.3%), lip mucosa/semimucosa (12.9%), buccal mucosa (8.6%), alveolar mucosa (8.6%), gingiva (5.7%), and floor of mouth (4.3%). Two participants had oral plexiform neurofibromas (Figure 1), associated with ipsilateral facial plexiform neurofibromas and hemimacroglossia; one progressed to malignant peripheral nerve sheath tumor.

Nine NF1 participants underwent removal of clinically suspected oral neurofibromas; histopathology confirmed 88%, the remainder being focal fibrous hyperplasia or fibrolipoma.

Oral neurofibromas were more common between ages 41 and 50, youngest case 22 years, and correlated positively with age (Spearman's *r* = 0.433; *p* < 0.0001). They were more frequent in those without a family history of NF1 (50% vs. 29%; *p* = 0.032, Pearson's chi‐square), though counts did not differ (*p* = 0.068, Mann–Whitney U test).

Cutaneous neurofibroma counts averaged 743.0 ± 821.4; those with oral neurofibromas had higher counts (1133.9 ± 900.6 vs. 489.4 ± 662; *p* < 0.0001, Mann–Whitney *U* test). Oral and cutaneous counts correlated (Spearman's *r* = 0.505; *p* < 0.0001). LOESS modeling showed ~300 cutaneous neurofibromas corresponded to ~2 oral neurofibromas and ≥ 1750 to > 2. Pregnancy history showed no association with oral neurofibroma presence (*p* = 0.564, Pearson's chi‐square) or count (Spearman's *p* = 0.276). Physiological melanin pigmentation was more frequent in NF1 (Table 2) and associated with Black participants (*p* < 0.0001, Pearson's chi‐square).

## 4 Discussion

This study revealed a high frequency of oral alterations in NF1, expanding current knowledge. Soft tissue alterations were present in 92.6% of NF1 individuals, exceeding previous reports (66%–74%) [2, 6]. Enlarged fungiform papillae were the most common finding and may serve as an early NF1 indicator, though their pathogenesis remains unclear. Our recent study found reduced sweet and sour taste perception and poor eating habits in NF1, with no association to papillae size [7].

Coated tongue, the second most common finding, showed no association with papillae enlargement and may relate to hyposalivation, diet, or hygiene. However, in a previous study we reported a 55% prevalence of hyposalivation, without direct association with coated tongue [8].

Oral neurofibromas occurred in 38.3% of NF1 individuals, similar to Jouhilahti et al. [2]. Multiple lesions were present in 17.3% of cases, reinforcing the need for regular monitoring. They were more frequent in participants without a family history of NF1, unlike previous findings for cutaneous neurofibromas, suggesting that family history may differently influence their development in skin and oral mucosa [9].

Oral neurofibroma counts correlated with cutaneous lesions, but skin tumors were more numerous, suggesting lower oral mucosa permissiveness. The palate was the most frequent site, unlike prior reports emphasizing the tongue [2, 6, 10], and may impair function, warranting regular evaluation. Unusual presentations, such as erythematous macules, may cause underdiagnosis.

Physiological pigmentation was more frequent in NF1, possibly reflecting altered neurofibromin function in melanocytes. While cutaneous pigmentations are well recognized [11], oral changes remain understudied.

Exostoses prevalence (12.3%) was slightly higher than the 9% reported by Shapiro et al. [6]. Neurofibromin expression in bone cells and high frequency of abnormalities in other bones (up to 70%) [12], suggest a possible association between oral exostoses and NF1, warranting further investigation.

Limitations of this study include unmatched groups, lack of blinding during evaluations, and retrospective design. Biopsies were not performed for all oral neurofibromas, and the onset age of oral neurofibromas was unclear.

In conclusion, oral alterations are frequent in NF1, with papillae enlargement occurring in younger patients and age‐related neurofibromas, which are associated with higher cutaneous burden and NF1 sporadic cases. Other common findings included coated tongue, physiological pigmentation, and oral exostoses. While some alterations may not require treatment, regular oral evaluations are essential for early detection of significant lesions, and hygiene promotion may help manage conditions like coated tongue.

## Author Contributions

**Pâmella de Pinho Montovani:** investigation, formal analysis, writing – original draft. **Gabriela Pizão Werneck Moreira da Costa:** investigation. **Rafaela Elvira Rozza‐de‐Menezes:** project administration, writing – review and editing and supervision, formal analysis. **Karin Soares Cunha:** project administration, writing – review and editing and supervision. All authors read and approved the final manuscript.

## Ethics Statement

This work was approved by the research ethics committee of HUAP (#4.780.584) and the procedures performed were in accordance with the Helsinki Declaration of 1975, as revised in 1983.

## Conflicts of Interest

The authors declare no conflicts of interest.

## Data Availability

The data that support the findings of this study are available from the corresponding author upon reasonable request.
